# Systems Biology Approaches to Mining High Throughput Biological Data

**DOI:** 10.1155/2015/504362

**Published:** 2015-08-04

**Authors:** Fang-Xiang Wu, Min Li, Jishou Ruan, Feng Luo

**Affiliations:** ^1^School of Mathematical Sciences, Nankai University, Tianjin 300071, China; ^2^Division of Biomedical Engineering, University of Saskatchewan, Saskatoon, SK, Canada S7N 5A9; ^3^School of Information Science and Engineering, Central South University, Changsha 410083, China; ^4^School of Computing, Clemson University, Clemson, SC 29634, USA

With advances in high throughput measurement techniques, large-scale biological data have been and will continuously be produced, for example, gene expression data, protein-protein interaction (PPI) data, tandem mass spectra data, microRNA expression data, lncRNA expression data, and biomolecule-disease association data. Such data contain insightful information for understanding the mechanism of molecular biological systems and have proved useful in diagnosis, treatment, and drug design for genetic disorders or complex diseases. For this focus issue, we have invited the researchers to contribute original research articles which develop or improve systems biology approaches to mining high throughput biological data.

With high throughput data, it is appealing to develop systems biology approaches to understand important biological processes. In the paper “Differential Expression Analysis in RNA-Seq by a Naive Bayes Classifier with Local Normalization,” Y. Dou et al. developed a new tool for the identification of differentially expressed genes with RNA-Seq data, named GExposer. This tool introduced a local normalization algorithm to reduce the bias of nonrandomly positioned read depth. The Naive Bayes classifier was employed to integrate fold change, transcript length, and GC-content to identify differentially expressed genes. Results on several independent tests showed that GExposer had better performance than other methods. In the paper “K-Profiles: A Nonlinear Clustering Method for Pattern Detection in High Dimensional Data,” K. Wang et al. designed the nonlinear* K*-profiles clustering method, which can be seen as the nonlinear counterpart of the* K*-means clustering algorithm. The method had a built-in statistical testing procedure that ensures genes not belonging to any cluster do not impact the estimation of cluster profiles. Results from extensive simulation studies showed that* K*-profiles clustering outperformed traditional linear* K*-means algorithm. In addition,* K*-profile clustering generated biologically meaningful results from a gene expression dataset.

Replicative senescence is of fundamental importance for the process of cellular aging. In the paper “Similarities in Gene Expression Profiles during In Vitro Aging of Primary Human Embryonic Lung and Foreskin Fibroblasts,” S. Diekmann et al. elucidated cellular aging process by comparing gene expression changes, measured by RNA-Seq, in fibroblasts originating from two different tissues, embryonic lung (MRC-5) and foreskin (HFF), at five different time points during their transition into senescence. Their results showed that a number of monotonically up- and downregulated genes had a novel strong functional link to aging and senescence related processes.

More and more studies have shown that many complex diseases are contributed jointly by alterations of numerous genes. Genes often coordinate together as a functional biological pathway or network and are highly correlated. In the paper “Module Based Differential Coexpression Analysis Method for Type 2 Diabetes,” L. Yuan et al. proposed a gene differential coexpression analysis algorithm and applied it to a publicly available type 2 diabetes (T2D) expression dataset. Two differential coexpression gene modules about T2D were detected and were expected to be useful for exploring the biological functions of the related genes.

Oral mucosa is a useful material for regeneration therapy with the advantages of its accessibility and versatility regardless of age and gender. In the paper “Gene Signature of Human Oral Mucosa Fibroblasts: Comparison with Dermal Fibroblasts and Induced Pluripotent Stem Cells,” K. Miyoshi et al. reported the comparative profiles of the gene signatures of human oral mucosa fibroblasts (hOFs), human dermal fibroblasts (hDFs), and hOF-derived induced pluripotent stem cells (hOF-iPSCs), linking these with biological roles by functional annotation and pathway analyses. Their findings demonstrated that hOFs had unique cellular characteristics in specificity and plasticity. These data may provide useful insight into application of oral fibroblasts for direct reprograming.

Predicting disease genes for a particular genetic disease is very challenging. However, this challenge can be tackled via exploring high throughput data. In the paper “ProSim: A Method for Prioritizing Disease Genes Based on Protein Proximity and Disease Similarity,” G. U. Ganegoda et al. proposed a new algorithm called proximity disease similarity algorithm (ProSim), which took use of two types of data: disease similarity data and protein-protein interaction data. The computational results have shown that their proposed method outperformed existing methods.

In order to learn the protein structures and functions via computational methods, it is important to predict the solvent accessibility and the contact number of protein residues from protein sequence. In the paper “AcconPred: Predicting Solvent Accessibility and Contact Number Simultaneously by a Multitask Learning Framework under the Conditional Neural Fields Model,” J. Ma and S. Wang presented a method AcconPred for predicting solvent accessibility and contact number simultaneously, which was based on a shared weight multitask learning framework under the CNF (Conditional Neural Fields) model. Trained on a 5729 monomeric soluble globular protein dataset, AcconPred could reach 0.68 three-state accuracy for solvent accessibility and 0.75 correlation for the contact number. Tested on the 105 CASP11 domain dataset for solvent accessibility, AcconPred could reach 0.64 accuracy, which outperformed existing methods.

The Smith-Waterman algorithm is one of the key sequence search algorithms for sequence alignment and has gained popularity due to improved implementations and rapidly increasing compute power. Recently, the Smith-Waterman algorithm has been successfully mapped onto the emerging general-purpose graphics processing units (GPUs). In the paper “Improving the Mapping of Smith-Waterman Sequence Database Searches onto CUDA-Enabled GPUs,” L.-T. Huang et al. employed the CUDA-enabled GPU to improve the mapping of Smith-Waterman algorithm, especially for short query sequences. The computational results showed that the proposed method significantly improved Smith-Waterman algorithm on CUDA-enabled GPUs in proper allocation of block and thread numbers.

Protein interaction article classification is a text classification task in the biological domain to determine which articles describe protein-protein interactions. In the paper “Improving Classification of Protein Interaction Articles Using Context Similarity-Based Feature Selection,” Y. Chen et al. proposed new context similarity-based feature selection methods. Their performances were evaluated on two protein interaction article collections and compared against the frequency-based methods. The experimental results revealed that the context similarity-based methods performed better in terms of the F1 measure and the dimension reduction rate.

Recent studies suggest that posttranscriptional RNA modifications play a crucial role in regulating gene expression. In practice, a single methylation site can contain multiple RNA methylation residuals, some of which can be regulated by different enzymes and thus differentially methylated between two conditions. However, existing peak-based methods could not effectively differentiate multiple methylation residuals located within a single methylation site. In the paper “Spatially Enhanced Differential RNA Methylation Analysis from Affinity-Based Sequencing Data with Hidden Markov Model,” Y.-C. Zhang et al. proposed a hidden Markov model (HMM) based approach to address this issue. The proposed algorithms were tested on both simulated data and real data. Results suggested that their proposed algorithm clearly outperformed existing peak-based approach on simulated systems and could detect differential methylation regions with higher statistical significance on real data, indicating an improved performance.

Pregnane X Receptor (PXR) and drug-metabolizing target genes are involved in most of inductive herb-drug interactions. To predict this kind of herb-drug interactions, the protocol could be simplified to only screen agonists of PXR from herbs because the relations of drugs with their metabolizing enzymes are well studied. In the paper “Screening Ingredients from Herbs against Pregnane X Receptor in the Study of Inductive Herb-Drug Interactions: Combining Pharmacophore and Docking-Based Rank Aggregation,” Z. Cui et al. employed a combinational in silico strategy of pharmacophore modelling and docking-based rank aggregation (DRA) to identify PXR's agonists. To validate their method, a curated herb-drug interaction database was built, which recorded 380 herb-drug interactions. The results showed that, among the top 10 herb ingredients from the ranking list, 6 ingredients were reported to involve herb-drug interactions.

In summary, this focus issue has reported the recent progress in systems biology approaches to analyzing high throughput data such as gene expression data, various biomolecular interaction data, and sequencing data. We hope that the readers of this focus issue could get some benefits from these newly developed methods.

*Fang-Xiang Wu* *Min Li* *Jishou Ruan* *Feng Luo*

